# Genetic diversity and evolutionary analysis of human respirovirus type 3 strains isolated in Kenya using complete hemagglutinin-neuraminidase (HN) gene

**DOI:** 10.1371/journal.pone.0229355

**Published:** 2020-03-10

**Authors:** Juliet Elusah, Wallace Dimbuson Bulimo, Silvanos Mukunzi Opanda, Samwel Lifumo Symekher, Fred Wamunyokoli

**Affiliations:** 1 Department of Biochemistry, Jomo Kenyatta University of Agriculture and Technology, Juja, Kenya; 2 Department of Emerging infections, US Army Medical Directorate–Africa, Nairobi, Kenya; 3 Department of Biochemistry, School of Medicine, University of Nairobi, Nairobi, Kenya; 4 Center for Virus Research, Kenya Medical Research Institute, Nairobi, Kenya; Loyola University Health System, UNITED STATES

## Abstract

Human respirovirus type 3 (HRV3) is a leading etiology of lower respiratory tract infections in young children and ranks only second to the human respiratory syncytial virus (HRSV). Despite the public health importance of HRV3, there is limited information about the genetic characteristics and diversity of these viruses in Kenya. To begin to address this gap, we analyzed 35 complete hemagglutinin-neuraminidase (HN) sequences of HRV3 strains isolated in Kenya between 2010 and 2013. Viral RNA was extracted from the isolates, and the entire HN gene amplified by RT-PCR followed by nucleotide sequencing. Phylogenetic analyses of the sequences revealed that all the Kenyan isolates grouped into genetic Cluster C; sub-clusters C1a, C2, and C3a. The majority (54%) of isolates belonged to sub-cluster C3a, followed by C2 (43%) and C1a (2.9%). Sequence analysis revealed high identities between the Kenyan isolates and the HRV3 prototype strain both at the amino acid (96.5–97.9%) and nucleotide (94.3–95.6%) levels. No amino acid variations affecting the catalytic/active sites of the HN glycoprotein were observed among the Kenyan isolates. Selection pressure analyses showed that the HN glycoprotein was evolving under positive selection. Evolutionary analyses revealed that the mean TMRCA for the HN sequence dataset was 1942 (95% HPD: 1928–1957), while the mean evolutionary rate was 4.65x10^-4^ nucleotide substitutions/site/year (95% HPD: 2.99x10^-4^ to 6.35x10^-4^). Overall, our results demonstrate the co-circulation of strains of cluster C HRV3 variants in Kenya during the study period. This is the first study to describe the genetic and molecular evolutionary aspects of HRV3 in Kenya using the complete HN gene.

## Introduction

Human parainfluenza viruses (HPIVs) are significant aetiologic agents of upper and lower respiratory tract infections (RTIs). The most susceptible groups to these viruses are children, the elderly, and immunocompromised persons [1, 2]. These viruses belong to the family *Paramyxoviridae* and are grouped into four types, HPIV 1–4, based on genetic and antigenic differences [3, 4]. HPIV types 2 and 4 are members of the genus *Rubulavirus* [5–8], whereas HPIV types 1 and 3 are classified as members of the genus *Respirovirus*. Thus, these two viruses are currently known as Human Respirovirus types 1 and 3 abbreviated as HRV1 and HRV3, respectively [5–8]. Each of these virus types is associated with distinct respiratory disease manifestations/clinical features [9]. Amongst the genus *Respirovirus*, HRV3 is the most frequent etiology of acute lower respiratory tract infection (such as pneumonia and bronchiolitis) in infants and young children, ranking second only to the human respiratory syncytial virus (RSV) [3, 10–12].

The HRV3 genome consists of a monopartite negative-sense single-stranded RNA molecule of approximately 15460 nucleotides. The genome encodes six viral proteins, namely the nucleocapsid protein (NP), phosphoprotein (P), matrix (M), fusion (F), hemagglutinin-neuraminidase (HN) and the large protein (L) [13]. The hemagglutinin-neuraminidase (HN) glycoprotein harbor neuraminidase and hemagglutinin activities [5]. The HN protein is involved in virus entry/budding and is believed to be the major virus surface antigen [14]. The HN protein recognizes and adsorbs to the host’s cell-surface α2,6 or α2,3 sialic acid receptors, initiating virus infection [15, 16]. The HN gene is antigenically and genetically highly variable, and therefore is a favorite target in studying molecular epidemiology of HRV3 [12, 15, 17].

Despite being a pathogen of great public health importance responsible for severe respiratory illnesses, especially among infants and children globally [13], information on the genetic diversity, phylogeny, and evolutionary characteristics of HRV3 viruses circulating in Kenya remain scanty. This information is crucial in guiding the development of molecular diagnostics and vaccines [18]. The present study, therefore, sought to elucidate the genetic diversity and evolution patterns of HRV3 viruses that circulated in Kenya between 2010 and 2013, a period when there were heightened incidences of infectious respiratory diseases in Kenya exacerbated by the pandemic influenza.

## Methods

### Virus isolates

The HRV3 isolates used in this study were first propagated in Rhesus Monkey Kidney cells (LLCMK2) (ATCC® CCL-7.1™) and presence of the virus confirmed by direct fluorescent antibody (DFA) test using Light Diagnostics™ SimulFluor® Parainfluenza 1, 2/3 Immunofluorescence Assay Kit (Millipore Corporation, USA) following the manufacturer's instructions. The isolates had previously been recovered from Nasopharyngeal swab specimens obtained from patients presenting with influenza-like illness (ILI) according to the WHO case definition [19].

The clinical samples were obtained from outpatients attending hospitals located across the country between 2010 and 2013. The hospitals comprise part of the Kenya human respiratory virus surveillance program network for the Kenya Medical Research Institute (KEMRI). The patient samples were processed at the laboratory as previously described [20].

### Ethical approval

This study protocol was reviewed and approved by the KEMRI Scientific and Ethics Review Unit (SERU) under approval number SSC# 3298. Patients consented, as previously described [21].

### RT-PCR and sequencing

Viral RNA was extracted from culture supernatants using the QIAamp Viral Mini Kit (Qiagen CA, USA) according to the manufacturer's instructions. The complete HN gene was amplified by RT-PCR using three sets of primers previously described [1].

The PCR amplicons were electrophoresed on a 2% agarose gel (Sigma-Aldrich Co., USA) stained in 2 μg/ml ethidium bromide (Sigma-Aldrich Co., USA) solution and visualized using the E-box gel documentation system (Vilber Lourmat, France). The amplicons were subsequently purified using Exonuclease I/Shrimp Alkaline Phosphatase (ExoSap-IT) enzyme (Affymetrix, USA) and sequenced on both strands on an automated 3500xL Genetic Analyzer (Applied Biosystems, USA) using the same primers. Cycle sequencing was performed using the Big Dye Terminator Cycle sequencing kit v3.1 (Applied Biosystems, USA).

### Sequence accession numbers

The HN sequences of HRV3 isolates reported in this study are available in GenBank (www.ncbi.nlm.nih.gov/genbank) under accession numbers: MN116649—MN116682 and MN116749.

### Phylogenetic relationships and evolutionary analyses

The nucleotide sequences of the Kenyan HRV3 HN genes were aligned with those of reference strains retrieved from GenBank using Muscle v3.8 software [22]. Phylogenetic reconstruction was performed by MrBayes v3.2 software [23] under the best fit HKY+G substitution model as predicted by the jModelTest software [24]. A Maximum Clade Credibility (MCC) tree using the Markov chain Monte Carlo (MCMC) method implemented in BEAST package v1.8.4 [25] was generated. BEAST was run for 100 million generations, sampling every 10,000 steps with a 10% burn-in under an uncorrelated relaxed molecular clock and Coalescent GMRF Bayesian Skyride demographic model combination. The convergence of BEAST runs was confirmed by effective sample size (ESS) values >200 for all the parameters using Tracer program v1.6 [26]. The MCC tree was summarized using Tree Annotator [27]. Both trees were visualized and annotated using Fig Tree™ version 1.4.3 [28]. Estimates of evolutionary divergence between and within clusters were conducted in MEGA6 [29] using Kimura 2 parameter [30].

### Selection analysis and prediction of N-glycosylation sites

Positively selected sites on the HN genes of Kenyan HRV3 strains were estimated using single likelihood ancestor counting (SLAC), fast and unconstrained Bayesian approximation (FUBAR), random effects likelihood (REL), mixed-effects model of evolution (MEME), and fixed effects likelihood (FEL) algorithms implemented in the Datamonkey web server (http://www.datamonkey.org) [31]. Codon sites were considered to be under positive selection if identified by two or more methods, with a p-value less than 0.05 for SLAC, FEL, & MEME or Bayes factor/posterior probability of more than 0.95 for REL and FUBAR [11, 14]. Prediction of potential N-glycosylation sites across the HN protein was performed using the online NetNGlyc 1.0 server [32]. A threshold score value of > 0.5 was regarded as suggestive of glycosylation.

## Results

### Patient characteristics

**Fig 1:**
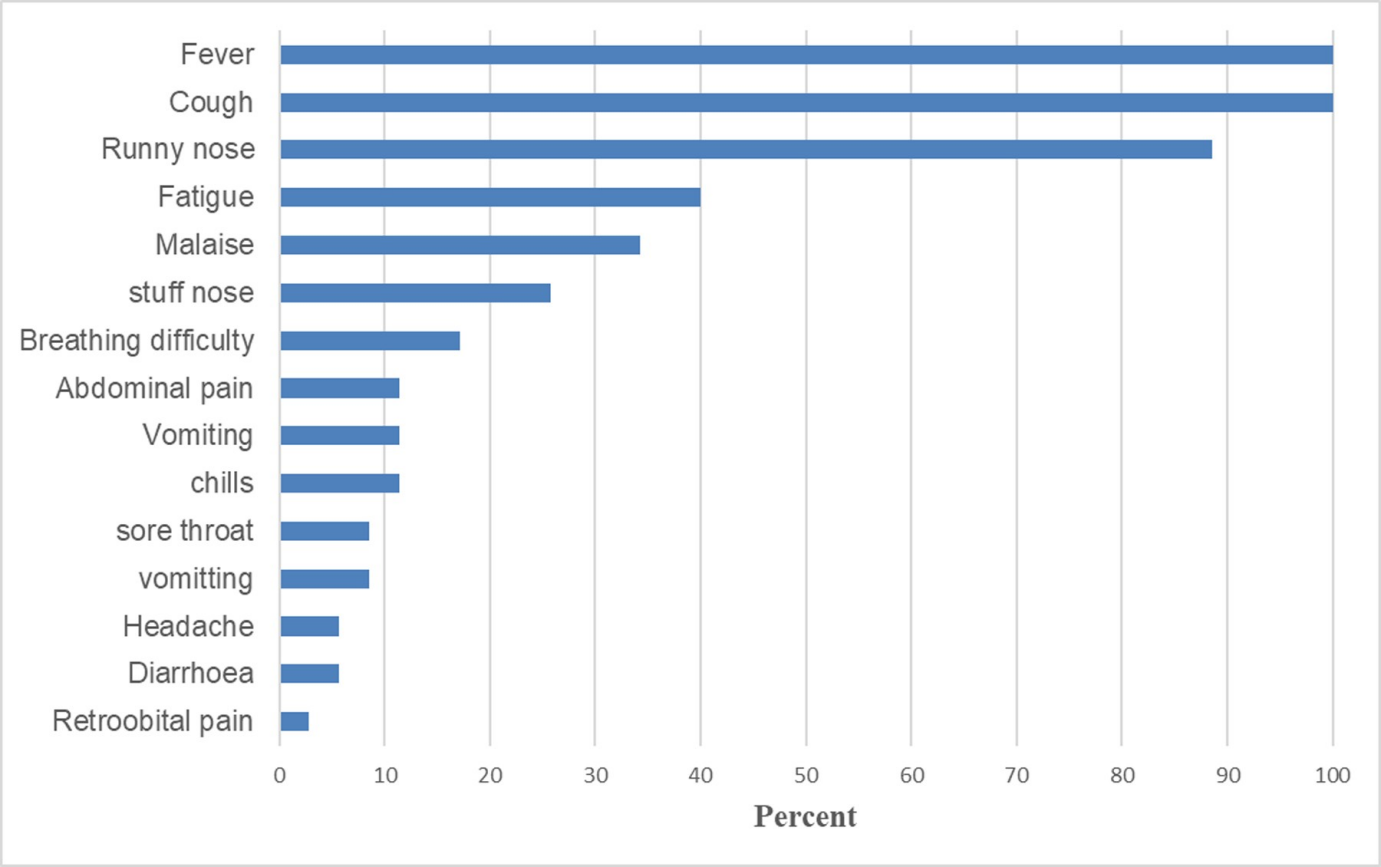
Summary of % distribution of presenting symptoms of patients with human respirovirus 3 infections in Kenya from June 2010 to June 2013.

All the 35 isolates from the surveillance program that had been confirmed to be positive for HRV-3 by the DFA antigen test were sequenced and analyzed in this study. The majority of the patients from whom the viruses were isolated were children (n = 34; 97.1%) and one adult (2.9%). The patients’ ages ranged from 3 months to 37 years. There were more male patients (n = 21; 60%) than female (n = 14; 40%). Among the patients enrolled, common signs and symptoms included fever (100%), cough (100%), runny nose (88.6%), malaise (42.85), fatigue (40%), stuffy nose (22.8%), chills and breathing difficulty (17%) or others including headache and sore throat (Fig 1).

### Positive selection and prediction of N-glycosylation sites

Overall, five codon sites on the HN gene of the Kenyan HRV3 viruses were shown to be under positive selection pressure by MEME and REL algorithms (Table 1). No positively selected sites were identified by either SLAC, FUBAR, or FEL algorithms. Prediction of N-glycosylation motifs across the HN protein revealed potential glycosylation at amino acid residue positions 308, 485, and 523 among the Kenyan HRV3 strains. However, these are also conserved in the HRV3 prototype strain (Accession number: JN089924).

**Table 1:** Positively selected sites by at least two algorithms (in bold) on the HN gene of Kenyan HRV3 strains.

Codon	SLAC (p-value)	FUBAR (Post Pr.)	MEME (p-value)	FEL (p-value)	REL (Post Pr.)
8	0.879	0.38	**0.0426**	0.273	**0.9998**
21	0.889	0.221	0.0116	0.407	N/A
33	0.89	0.225	0.0184	0.412	N/A
45	0.813	0.674	**0.029**	0.351	**0.9989**
66	0.517	0.784	0.0711	1	0.9653
168	0.708	0.751	**0.0263**	1	**0.9993**
191	0.708	0.742	**0.0408**	0.757	**0.9999**
555	0.43	0.816	**0.0108**	0.972	**0.9995**

### Genetic analyses and phylogenetic relationships

**Fig 2:**
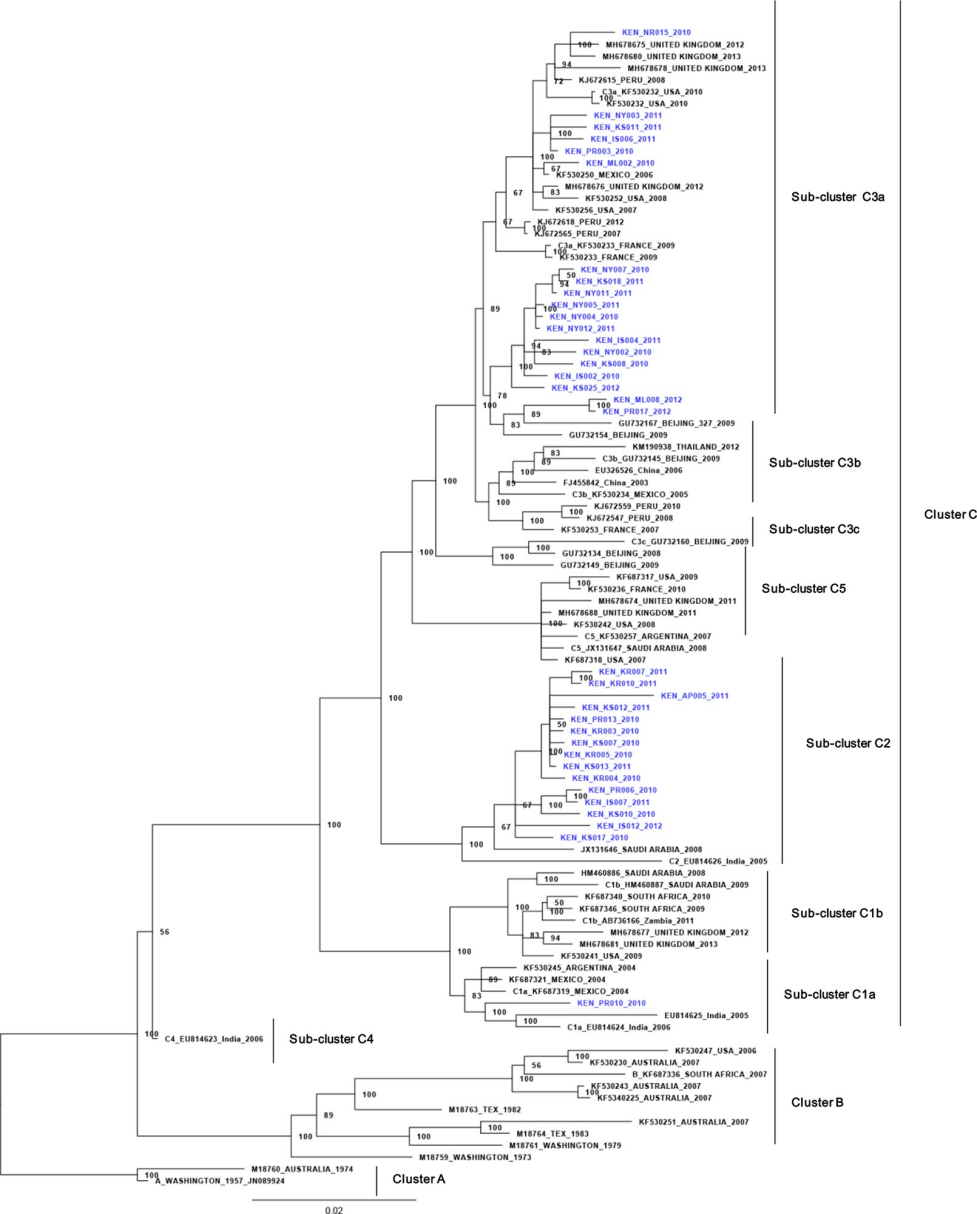
Bayesian phylogenetic tree based on nucleotide sequences of the complete HN gene of HRV3 strains. The tree was constructed using MrBayes v3.2. The numbers at the nodes represent posterior probability values. The scale bar indicates the number of nucleotide substitutions per site. Kenyan isolates are shown in blue.

Homology analyses of the complete HN gene sequences revealed that the Kenyan HRV3 viruses shared 94.3–95.6% (Nucleotide) and 96.5–97.9% (amino acid) identities with the prototype strain. Sequence alignment with the prototype strain showed that all the Kenyan viruses were conserved at the antigenic region of the HN protein. Phylogenetic comparisons of the Kenyan HRV3 viruses with global strains in GenBank revealed the delineation of the viruses into three main clusters A, B, and C (Fig 1). Cluster A contained the oldest HRV3 virus strain (prototype strain) from North America and an Australian strain, while Cluster B had variants from Australia, the United States of America, and a South African strain (Fig 2).

Furthermore, the strains belonging to Cluster C segregated into sub-clusters C1a, C1b, C2, C3a, C3b, C3c, C4 and C5 (Fig 2). All the Kenyan viruses grouped into cluster C among strains from across the globe with strong statistical support (posterior probability value 100%) and were distributed in sub-clusters C3a (54%), C2 (43%) and C1a (2.9%), (Fig 2). The Kenyan isolates belonging to sub-clusters C2 and C1a were closely related to viruses isolated in India and Saudi Arabia, while those belonging to sub-cluster C3a were highly similar to HRV3 viruses that circulated in Beijing, China and Mexico. Genetic distances (P) over sequence pairs between the phylogenetic clusters A:B, A:C, and B:C were calculated as 0.050, 0.137, and 0.142, respectively. The genetic distances between sequence pairs of Kenyan HRV3 strains and those of global reference strains in clusters A, B, and C were estimated at 0.056, 0.063, and 0.025, respectively. The lower genetic distance of the Kenyan viruses with the global strains in the C cluster confirmed that the HRV3 strains detected in the study indeed belonged to Cluster C. The genetic distances between the various sub-clusters within the cluster C ranged from 0.013 to 0.039.

### Time scale phylogenetic analysis and estimation of the evolutionary rate

**Fig 3:**
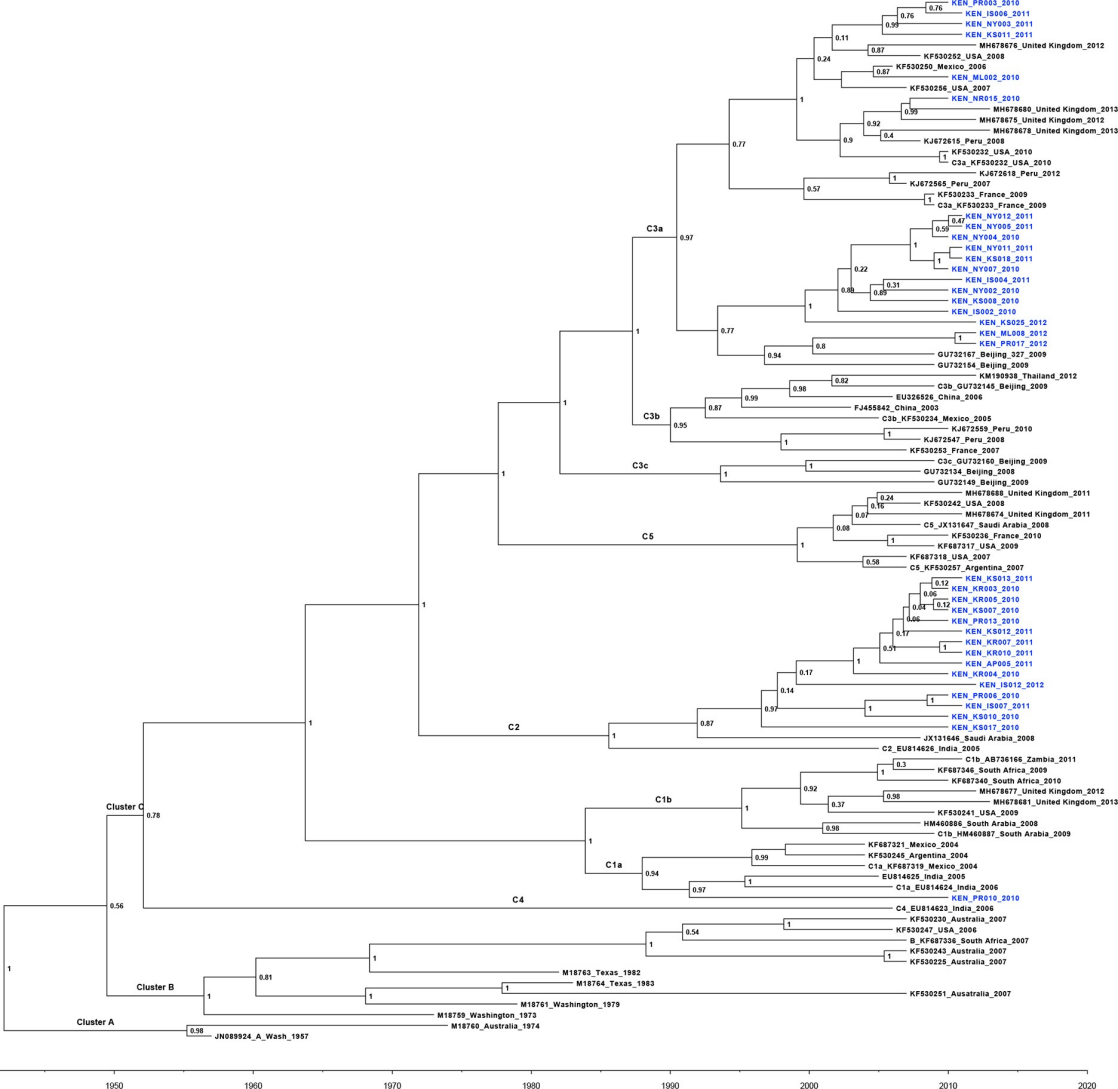
Maximum Clade Credibility (MCC) tree of HRV3 strains based on complete HN coding sequences, constructed using the BEAST program. The Kenyan strains are shown in blue. The numbers at the nodes represent posterior probability values.

Molecular evolutionary analysis using the BEAST software further established that the Kenyan HRV3 isolates belonged to Cluster C (high posterior probability value 100%), sub-clusters C3a (54%), C2 (43%) and C1a (2.9%) (Fig 2). The mean TMRCA of HRV3 for the whole sequence dataset was estimated at 1942 (95% HPD: 1928–1957) (Fig 3) while the mean evolutionary rate was 4.65x10^-4^ nucleotide substitutions/site/year (95% HPD: 2.99x10^-4^ to 6.35x10^-4^). Furthermore, our analyses indicated that the HRV3 virus infections in Kenya between 2010–2013 resulted from different introductions from various countries including India (Kenyan Sub-cluster C1a virus strains), Saudi Arabia (Kenya Sub-cluster C2 virus strains) and Beijing & the United States of America (Kenyan Sub-cluster C3a virus strains) (Fig 2).

## Discussion

This study described the genetic diversity and evolutionary analyses of HRV3 strains isolated in Kenya between 2010 and 2013, focusing on the HN glycoprotein. HRV3 has been associated with acute respiratory infection in children and aged persons, with the former group being most susceptible [30]. Indeed, per previous studies [29, 33–35] and our findings in this study, the majority of patients presenting with HRV3 infection are children aged between 3 months and two years. In addition, consistent with findings of Mizuta *et al*., and Košutić-Gulija *et al*., [14, 29], but contrasting Mao *et al*., and Counihan *et al*. [36, 37], majority of the patients in this study presented with symptoms commonly associated with upper respiratory infection such as fever, cough, and runny nose. It was interesting to note that none of the patients in this study presented with lower respiratory symptoms such as pneumonia and bronchitis, which are commonly associated with HRV3 infection [30, 38]. This observation may be attributed to the fact that our study enrolled only outpatients with ILI symptoms and not SARI hospitalized patients [29]. The ILI case definition excluded many of the potential cases, and this may explain why this study was able to detect only thirty-five patients with HRV3.

Phylogenetic analysis based on the complete HN gene sequence data revealed delineation of the HRV3 strains into three main clusters (A, B, and C) and all the Kenyan isolates grouped into phylogenetic cluster C, sub-clusters C3a, C2, and C1a. This result is consistent with previous findings showing that globally, the recently circulating HRV3 viruses all belong to cluster C [10, 29, 30, 38]. As previously reported [10, 12], cluster A comprised the HRV3 prototype strain and an Australian strain isolated in 1973, while cluster B contained a blend of old and recent strains isolated between 1974 and 2007, mostly in the United States and Australia. All the Kenyan viruses classified into cluster C with sub-cluster C3a being most prevalent in Kenya; this is consistent with global circulation trends of HRV3 [13, 36, 39, 40]. Indeed, genetic distance analyses confirmed that the Kenyan HRV3 was closely related to the viruses in cluster C compared to those in Cluster A. This finding is consistent with that from a previous study [41], which could explain the clustering pattern observed in this study. The HRV3 Cluster C variants have been reported to be well distributed around the globe in recent times [10, 13]. The grouping of Kenyan isolates into sub-clusters 3Ca, C2, and C1a, demonstrate co-circulation of multiple lineages of cluster C variants in the country, during the study period. Indeed, phylogenetic inferences reveal that these HRV3 variants were imported into Kenya at different time points from various source countries. Initially, the C1a viruses spread from Kenya into India in 1991, while the C2 variants were seeded into Kenya from Saudi Arabia in early 1991. The C3a viruses were imported into Kenya at two different time points. The first one in late 1996 from China followed later in early 2002 from the United States of America. Overall, our observations reveal the co-circulation of multiple lineages during this time in Kenya and are consistent with related studies reported previously [11, 12].

Molecular analyses of the data revealed a conserved HRV3 HN glycoprotein among the Kenyan isolates relative to the prototype strain. Sequence homology analysis showed that the Kenyan isolates were similar to the HRV3 prototype strain, both at the nucleotide and amino acid levels. Our result corroborates findings by Mizuta *et al*., [14] showing that the HRV3 virus is quite conserved in the HN glycoprotein. However, in contrast to a section of findings reported here [14], sporadic amino acid substitutions of no antigenic significance were observed in the HN protein of the Kenyan isolates. Amino acid residue variations in the catalytic or active sites of HRV3 HN glycoprotein may alter its function rendering the protein to be inactive, leading to unviable progeny [14]. Hence, amongst all the Kenyan strains, no amino acid changes were observed at these sites. Furthermore, consistent with the above observations, all the potential N-glycosylation motifs detected in the HN glycoprotein of the prototype HRV3 strain were conserved in the Kenyan isolate. Mutation at N-linked glycosylation sites of the HRV3 HN glycoprotein has been shown to impede receptor recognition and cleavage of the virus fusion protein (F protein) [38, 42], implying that the Kenyan strains remain very fit. Five codons involving the amino acid changes N8R, S45L, K168R, V191I and L555S were shown to be under positive selection within the HN glycoprotein of the Kenyan isolates. These were identified by both MEME and REL methods, with strong statistical support (MEME p-value < 0.05 and REL posterior probability > 0.95. The detection of amino acid residue L555S on the HN gene of the HRV3 as being positively selected by both MEME and REL algorithms has been reported elsewhere [30]. The inferred positively selected sites in our study did not fall into any of the antigenic/ active sites; therefore, they may not affect the function of the HN protein. This finding may further indicate the conserved nature of the HN glycoprotein. The failure by other algorithms used in this study to detect positively selected sites with strong evidence of statistical support may be attributed to the small analyzed sequence dataset.

The mean TMRCA of HRV3 HN glycoprotein for the whole sequence dataset was estimated at 1942 (95% HPD: 1928–1957) while the mean evolutionary rate was 4.65x10^-4^ nucleotide substitutions/site/year (95% HPD: 2.99x10^-4^ to 6.35x10^-4^). These results are similar to the findings by Linster *et al*., [3] and Bose *et al*. [11], respectively. The TMRCA of 1942 suggests that the HRV3 virus was already an established human pathogen way before its discovery in the late 1950s [5]. The estimated evolutionary rate is moderate, signifying that, were a vaccine formulation to be based on the prototype strain’s HN protein, it may be efficacious against the Kenyan viruses and hence appropriate for use in Kenya.

This study had some limitations. Since it relied on archived isolates, recovery of some HRV3 strains in culture may have failed, introducing bias in the HRV3 variants reported in the study. Secondly, since we used ILI instead of the SARI case definition in this study, we missed cases with severe presentations, including bronchitis and pneumonia. The ILI case definition resulted in low detections of HRV3. Lastly, the utilization of only the complete HN genes instead of whole genomes may fail to provide comprehensive insights into the genetic diversity and other evolutionary dynamics of HRV3 strains circulating in Kenya.

Notwithstanding these limitations, we have shown that HRV3 strains that circulated in Kenya during the study period classified into phylogenetic cluster C; sub-clusters C3a, C2, and C1a and were seeded into the country from multiple locations. Furthermore, we have confirmed a conservation of the HN protein among the HPIV-3 viruses, suggesting that it is a probable target for the development of vaccine and other therapeutics. In future, whole-genome studies would provide comprehensive insights into the genetic and evolutionary properties of HRV3 in Kenya.
